# Personality Traits and Mediating Pathways to Mortality Risk: A Systematic Review

**DOI:** 10.1037/hea0001335

**Published:** 2023-11-30

**Authors:** Christopher S. Grogan, Nicholas A. Turiano, Andrea Habenicht, Máire McGeehan, Páraic S. O’Súilleabháin

**Affiliations:** 1 Department of Psychology, University of Limerick; 2 Health Research Institute, University of Limerick; 3 Department of Psychology, West Virginia University

**Keywords:** mortality, personality, health, Five-Factor Model, mediation

## Abstract

**Objective:**

Personality traits have been regularly linked with all-cause mortality risk. However, what mechanisms may provide an indirect pathway from personality traits to mortality is unclear. We sought to systematically review the literature and provide an overview of the potential mechanisms that have been identified in the literature.

**Method:**

Five electronic databases (PubMed, Web of Science, CINAHL, PsycInfo, and PsycArticles) were searched from inception to January 27, 2023. From 611 studies initially identified, seven studies met the final inclusion criteria. These seven papers have a combined sample of 60,104 individuals (*M* = 8,585, *SD* = 14,600; range 957–44,094).

**Results:**

These papers found that several variables, such as smoking, inflammation biomarkers, blood pressure, and sleep, mediated the relationship between various personality traits and mortality. There was considerable variation in the impact of results across cohorts, even when looking at similar variables, and notable differences in methodological approaches and reporting were discussed.

**Conclusions:**

This review identified a small pool of research looking at a range of indirect pathways (mediating variables). The review identified traits with well-established associations with mortality risk, such as neuroticism, do not have consistent findings in the mediation literature and a high level of variance in the degree to which mediators account for the personality–mortality relation between different cohorts. Despite these limitations, it is clear that examining indirect effects (mediation) has a crucial role to play in developing our understanding of the complex pathways that connect personality–mortality risk. We identify several avenues and considerations for future research.

## RESUMEN

**Objetivos:**

Los rasgos de personalidad se han relacionado regularmente con el riesgo de mortalidad por todas las causas. Sin embargo, no está claro qué mecanismos pueden proporcionar una vía indirecta de los rasgos de personalidad a la mortalidad. Intentamos revisar sistemáticamente la literatura y proveer una descripción general de los mecanismos potenciales que se han identificado en la literatura.

**Métodos:**

Se realizaron búsquedas en cinco bases de datos electrónicas (PubMed, Web of Science, CINAHL, PsycInfo, y PsycArticles) desde el inicio hasta el 27 de enero de 2023. De 611 estudios identificados inicialmente, siete estudios cumplieron con los criterios de inclusión finales. Estos siete artículos tienen una muestra combinada de 60,104 personas (*M* = 8,585, *DS* = 14,600; rango 957–44,094).

**Resultados:**

Estos artículos encontraron que algunas variables, como el tabaquismo, los biomarcadores de inflamación, la presión arterial y el sueño, mediaban la relación entre diversos rasgos de personalidad y la mortalidad. Hubo una variación considerable en el impacto de los resultados entre las cohortes, incluso cuando se analizaron variables similares y se discutieron diferencias notables en los enfoques metodológicos y en los informes.

**Conclusiones:**

Esta revisión identificó un pequeño grupo de investigaciones que examinan una variedad de mediadores. La revisión identificó que rasgos con asociaciones bien establecidas con el riesgo de mortalidad, como el neuroticismo, no tienen hallazgos consistentes en la literatura sobre mediación y un alto nivel de variación en el grado en que explican la relación personalidad-mortalidad entre diferentes cohortes. A pesar de estas limitaciones, está claro que examinar los efectos indirectos desempeña un papel crucial en el desarrollo de nuestra comprensión de las complejas vías que conectan la personalidad con el riesgo de mortalidad. Identificamos varias vías y consideraciones para futuras investigaciones.

Personality traits have been identified as predictors of important health outcomes such as mortality risk (Chapman et al., 2011; Strickhouser et al., 2017). Research utilizing the traits from the Five-Factor Model of Personality (Graham et al., 2017; O’Súilleabháin et al., 2021) has consistently found higher conscientiousness to be associated with a reduced mortality risk (Bogg & Roberts, 2013; Jokela et al., 2013; Kern & Friedman, 2008). While higher levels of neuroticism are typically associated with increased mortality risk (Chapman et al., 2011; Lahey, 2009; O’Súilleabháin & Hughes, 2018; Weiss & Costa, 2005; Wilson et al., 2005), notable exceptions to this have also been observed including papers finding no associations (Friedman et al., 1995; Jokela et al., 2013) and those finding that neuroticism is associated with lower mortality risk (Weiss & Costa, 2005; Weiss et al., 2013). While more mixed, some evidence exists for the remaining traits from the Five-Factor Model being associated with mortality risk. Higher openness (Ferguson & Bibby, 2012) and the subfacet of creativity (Turiano, Spiro, & Mroczek, 2012) are associated with a decreased mortality risk. Graham et al. (2017) note that in younger cohorts, openness is associated with increased levels of smoking but finds no significant relationship between openness and mortality risk. Similarly, papers have had a range of findings related to extraversion identifying it as being associated with increasing mortality risk (Ploubidis & Grundy, 2009), reducing mortality risk (Masui et al., 2006), or more commonly having no significant impact (Jokela et al., 2013; O’Súilleabháin & Hughes, 2018).

Broadly, the lifespan perspective provides numerous avenues to conceptualize the way in which personality may impact mortality across the life course. Both critical period models (consequences of exposure to risk during critical periods have longer-lasting effects than at other times) and accumulative models (impact of exposure to risk accumulates across life) are useful to understand how personality may impact longevity (Baltes & Goulet, 1970, Hampson & Friedman, 2008). More specific models have been proposed to conceptualize the mechanisms between personality and health outcomes. Smith (2006) outlines several models that posit possible pathways for how personality may influence health outcomes. These models focus on how personality influences things like health behaviors, stress appraisal, physiological factors, and coping methods. Friedman and Kern (2014) have emphasized the need for elaborate models accounting for multiple variables and the need to test these models empirically to develop a coherent understanding of the personality–health relationship. An explanatory model developed by Turiano et al. (2021), the Personality and Health Processes Model, also attempts to capture an integrated model of how personality can influence health outcomes through multiple mechanisms simultaneously. Although the research linking personality and health outcomes has been growing, as well as advancements in theoretical foundations for why personality predicts health, empirical evidence for the mechanisms underlying this relation are still lacking. Furthermore, given the huge variety of ways personality likely impacts health, it is challenging to clearly understand which pathways may be viable options in the personality–mortality relation.

Personality traits are associated with a broad range of health-related processes and outcomes, such as the tendency to smoke (Mroczek et al., 2009), aerobic capacity (Terracciano et al., 2013), drug use (Terracciano et al., 2008), self-rated physical health (Turiano, Pitzer, et al., 2012), cholesterol (Sutin, Terracciano, Deiana, Naitza, et al., 2010), inflammatory biomarkers (Sutin, Terracciano, Deiana, Uda, et al., 2010), cognitive health (Sutin et al., 2022), and risk of developing Alzheimer’s disease, dementia, and cardiovascular disease (Aschwanden et al., 2022; Terracciano et al., 2021). While personality traits are linked to a broad range of health-related factors, it is still unclear which factors provide an indirect (mediating) pathway linking these traits to future mortality risk. Fortunately, these questions can now be examined with advances in time-to-event analysis within a structural equation modeling framework.

Taken together, with the effect of personality traits on mortality risk being similar in magnitude to those of socioeconomic status (Roberts et al., 2007), it is critically important to uncover the mechanisms through which these effects are being realized and move from theoretical theories as to why personality influence mortality to a more robust understanding of the underlying mechanisms. This evidence base may then be able to identify individual differences in health outcomes and provide support for public health interventions (Bogg & Roberts, 2013). As such, this article aims to identify, synthesize, and critically evaluate the research examining indirect pathways linking personality traits to future mortality risk.

## Method

### Transparency and Openness

This review adheres to the Preferred Reporting Items for Systematic Reviews and Meta-Analyses (PRISMA) guidelines (Page et al., 2021).

### Eligibility Criteria

Papers were eligible for inclusion in the review if they met the following criteria:

1. Included participants who completed a personality trait measure (e.g., NEO-PI).
2. Recorded all-cause mortality as a primary outcome.
3. Assessed the personality–mortality relationship utilizing hazard ratios (HR), odds ratios, or risk estimate methods.
4. Papers must be cohort studies and must look at individual-level data.
5. Included a mediating variable exploring the relationship between personality traits and mortality.

Papers were excluded from the review if either of these criteria applied:

1. Published in a language other than English.
2. Not peer-reviewed.

### Search Strategy and Screening

A systematic literature review was conducted to capture all currently available empirical papers investigating the personality/mortality relationship. The search strategy combined terms for the personality traits (neuroticism, extraversion, openness, agreeableness, and conscientiousness), with references to the “Big-5,” construct or tools used to measure it along with common variations in spelling (Big 5, Big Five, NEO Personality Inventory, NEO-PI, Eysenck Personality Inventory, etc.), and common synonyms for mortality in the research literature (longevity, death). See the online supplemental materials for the complete search strategy. The exact search string syntax and format were tailored to each database.

The search was run across five databases (PubMed, Web of Science, CINAHL, PsycInfo, and PsycArticles) on January 27, 2023. These databases were selected based on coverage of key journals of interest, which were identified during preliminary reading before commencing the review. Abstracts were screened for eligibility by the primary author (Christopher S. Grogan), with a second researcher (Andrea Habenicht) reviewing a randomly selected 50% of the papers. This was followed by a full-text screening based on the abovementioned eligibility criteria. The screening was completed with the Rayyan Intelligent Systematic Review web tool (Ouzzani et al., 2016), thus allowing reviewers to screen papers in an independent blinded way before coming together to resolve conflicts. Any conflicts resulting from this screening process were jointly reviewed, and a consensus decision was made. A further detailed manual search was carried out using snowball methodology, looking at three sample papers identified as likely candidates for inclusion after abstract screening (O’Súilleabháin et al., 2021; Turiano, Hill, et al., 2012; Turiano et al., 2015). References included in these three papers and those that included them as a reference (as of January 27, 2023) were reviewed for possible inclusion.

### Data Extraction and Risk of Bias

Following paper extraction for inclusion, data were extracted across a set list of criteria. Risk of bias analysis was completed across all extracted papers using the Critical Appraisal Skills Programme (CASP) cohort study checklist (CASP, 2018). The second reviewer (Andrea Habenicht) then reviewed half of the data extracted and half of the risk of bias checklists to ensure the accuracy of information.

## Results

### Literature Search and Selected Papers

**Figure 1:**
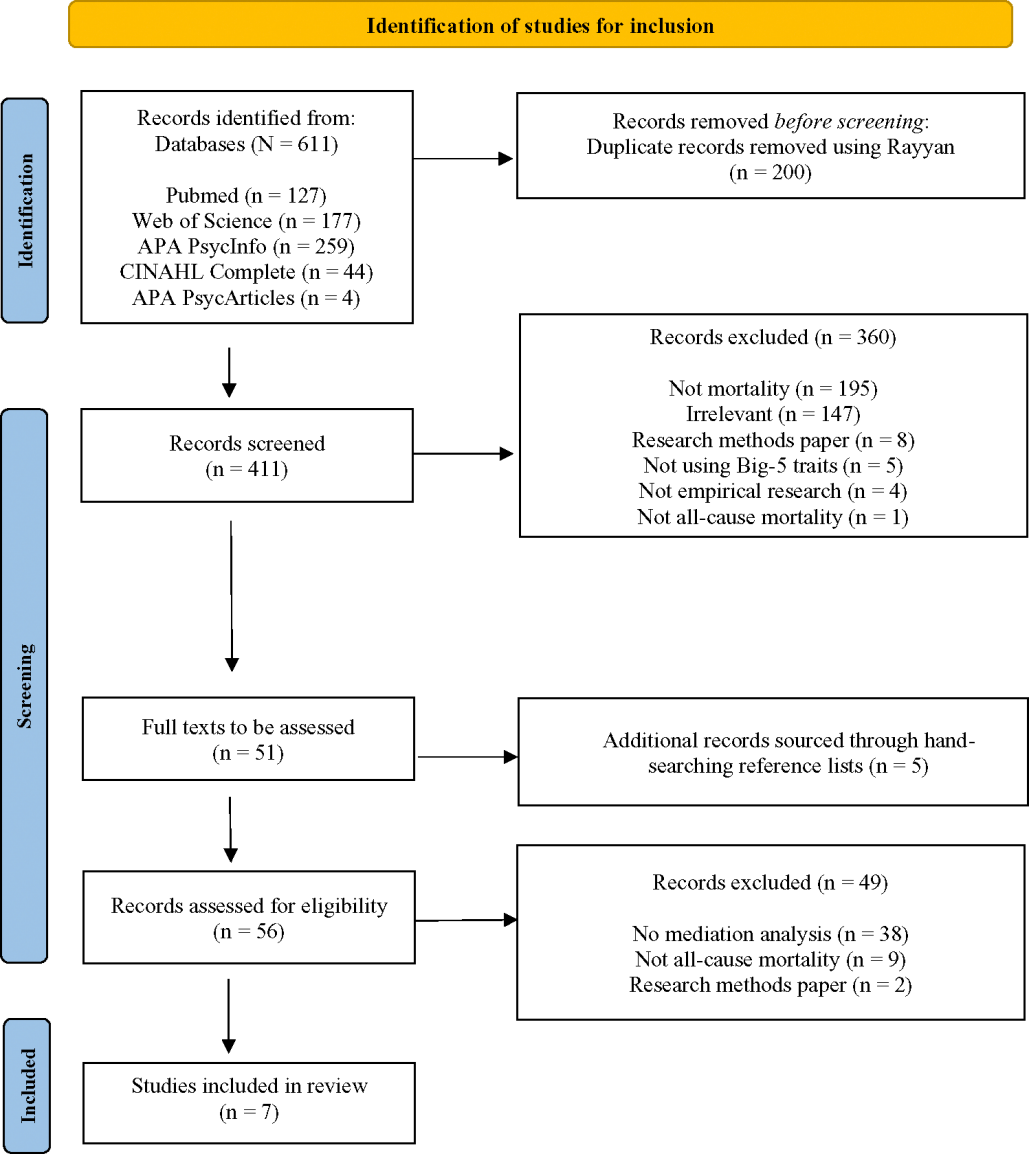
Flow Diagram of Articles Selected for Systematic Review *Note*. See the online article for the color version of this figure.

Figure 1 summarizes the screening procedure for included studies. The digital search resulted in 311 nonduplicate publications, and a further five possible papers were identified through a manual review of references and citations of three eligible papers (O’Súilleabháin et al., 2021; Turiano, Hill, et al., 2012; Turiano et al., 2015). Following the abstract screening, the full texts of 56 studies were screened. Resulting in a final selection of seven papers for inclusion in the review. The primary reason for exclusion in the abstract screening stage was that the papers did not focus on mortality as an outcome measure (*k* = 145) or were irrelevant to the present review (*k* = 97). During the full-text screening, most papers were excluded as they did not conduct mediation analysis (*k* = 38 papers).

### Quality of Included Papers

Quality checks of each paper were performed utilizing CASP cohort checklists (CASP, 2018). All seven papers identified for inclusion met the basic criteria of addressing a focused issue and utilizing acceptable methods in recruiting their cohorts. Full checklists with notes for each paper are in the online supplemental materials.

### Overview of Studies

Table 1 outlines a summary of the characteristics of included studies. The selected papers utilized data from the Edinburgh Artery Study (EAS), the Veterans Affairs Normative Aging Study (VA-NAS), the Swedish National Study of Aging and Care in Kungsholmen (SNAC-K), Midlife in the United States Study (MIDUS), and an integrated data analysis combining 15 samples stemming from a variety of databases from the Integrative Analysis of Longitudinal Studies on Aging (IALSA) network including Wisconsin Longitudinal Study Grads and Sibs (WLS), MIDUS, NAS, and the Health and Retirement Study (HRS). These study populations originated from the United Kingdom, Sweden, Australia, the Netherlands, and the United States. The seven papers included in this review have a combined sample of 60,104 individuals (*M* = 8,585, *SD* = 14,600; range 957–44,094). It should be noted here that the large range in sample size is due to one of the studies (Graham et al., 2017), a coordinated data analysis involving several data sets.

### Measurement of Variables and Data Analysis

#### Personality Measurement

All papers measured personality traits from the Five-Factor Model of Personality, namely neuroticism, extraversion, openness, agreeableness, and conscientiousness. Three of the papers used the Midlife Development Inventory (O’Súilleabháin et al., 2021; Spears et al., 2019; Turiano et al., 2015), two used the NEO Five-Factor Inventory (Rizzuto et al., 2017; Taylor et al., 2009), and one used Goldberg adjectival markers (Goldberg, 1992). Graham et al.’s (2017) research included data sets using a range of personality measures, NEO (*n* = 4), Eysenck (*n* = 4), scales derived from the International Personality Item Pool (*n* = 3), Goldberg’s adjective markers (*n* = 1), Dutch Personality Questionnaire (Eigenhuis et al., 2013; *n* = 1), Big Five Inventory (John et al., 1991; *n* = 2). The questionnaires used in this research body range from 25 to 100 items. Research examining the differences between the predictive power of different measures has found that shorter measures (.24 items) are as valid and functional for research when compared with medium-item measures (Thalmayer et al., 2011). All six measures used across the seven papers are valid assessments of Big-5 personality traits and have been shown to have high correlation coefficients between measures (Barelds & Luteijn, 2002; Goldberg, 1992; Lachman & Weaver, 1997; Soto & John, 2009). However, the majority of the reviewed studies utilized short form measures of personality. Such finer-grained facet-level trait measures might be key to uncovering specific personality–health associations (Butler et al., 2023; Danner et al., 2021; O’Súilleabháin et al., 2019; Paunonen & Ashton, 2001). The inclusion of these more comprehensive measures such as the NEO Personality Inventory Revised (Costa & McCrae, 2008), would provide opportunities for these analyses.

#### Mortality Measurement

All papers gathered mortality data from national records, with some papers also gathering information from participants’ general practitioners or families through closeout interviews (O’Súilleabháin et al., 2021; Spears et al., 2019; Taylor et al., 2009; Turiano, Hill, et al., 2012). Papers reported duration to death (or censorship) in the units of days (Taylor et al., 2009; Turiano, Hill, et al., 2012) or months (Graham et al., 2017; O’Súilleabháin et al., 2021; Rizzuto et al., 2017; Spears et al., 2019; Turiano et al., 2015). All the papers measured mortality as the time to death, defined as the period between when personality data were collected and the date of death.

### Reporting of Statistical Models

The models in the present sample controlled for a range of confounding variables for well-known influences on mortality. The most frequently included controls were age (*k* = 7), gender (*k* = 6), and education (*k* = 5). Some (*k* = 4) also adjusted for all other Big-5 personality traits in their regression models (O’Súilleabháin et al., 2021; Spears et al., 2019; Taylor et al., 2009; Turiano et al., 2015). Of the remaining papers, Rizzuto et al. (2017; *k* = 1) reported models both with and without the remaining traits, Turiano, Hill, et al. (2012; *k* = 1) focused on one trait, and Graham et al. (2017; *k* = 1) examined each separately followed by a sensitivity analysis to examine potential trait interactions. The papers each included covariates including marital status (*k* = 2; Spears et al., 2019; Turiano et al., 2015), self-rated health (*k* = 1; Turiano, Hill, et al., 2012), diagnosed chronic conditions (*k* = 1; O’Súilleabháin et al., 2021) and relevant medications (*k* = 1; O’Súilleabháin et al., 2021). In one paper (Taylor et al., 2009), the statistical analysis differs from the other papers; it conducts separate models for men and women and includes age, social class, smoking ( pack years), systolic blood pressure, and body mass index (BMI) both in Cox regressions and as independent variables in their structural equation models (SEM) rather than presenting them as covariates or adjustments.

### Mediation Analysis

**Figure 2:**
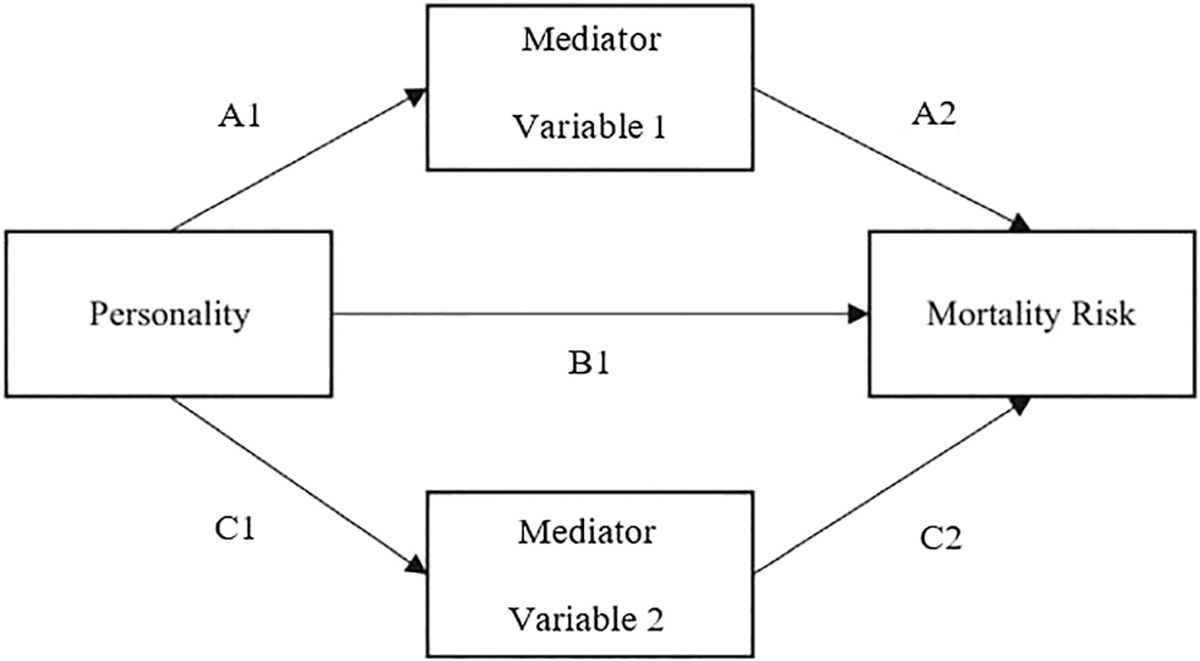
Example Mediation Model *Note*. Arrows and identifiers (e.g., A1) represent individual pathways in the relation between variables.

All papers included in the review used proportional hazard modeling in an SEM framework to estimate the direct and indirect effects on survival time. See Figure 2 for a graphical representation of this aproach. This approach computes the ratio of the path from predictor to mediator (A1) and the path from the mediator to the outcome (A2). It also assesses the direct effect of the predictor on the outcome variable (B1). The SEM analysis enables the assessment of both direct and indirect effects of individual personality traits on mortality outcomes through variables identified as potential mediators. The model can include multiple mediation and control variables. Mediation analysis was carried out using a range of software tools, including Mplus (Muthén & Muthén, 2017) Versions 6, 7.2, and 8 (Graham et al., 2017; O’Súilleabháin et al., 2021; Spears et al., 2019; Turiano, Hill, et al., 2012; Turiano et al., 2015), EQS 6 Structural Equations Program (Bentler, 1995; Taylor et al., 2009), and Stata V14.1 (Rizzuto et al., 2017; StataCorp, 2015).

#### Key Findings/Main Effects

The papers within this review found several personality traits that predicted all-cause mortality risk. In line with previous research, conscientiousness influenced the risk of all-cause mortality across all the papers. Other personality traits had less consistent findings; openness linked to an increase in the risk of mortality in Spears et al.’s (2019) paper and lower mortality risk in Taylor et al.’s (2009). Extraversion was associated with protective effects in two papers (Graham et al., 2017; Rizzuto et al., 2017), and agreeableness was associated with protective effects in one paper (Graham et al., 2017). Graham et al.’s (2017) paper was the only paper to find significant effects for neuroticism, with higher levels associated with an increased mortality risk. This finding was unexpected as neuroticism is frequently implicated within the personality–health literature.

Following the initial direct effect examination, all papers conducted SEM-based mediation analysis using various variables. These included health behaviors (e.g., smoking), physical assessments (e.g., waist circumference, BMI), and biological markers (e.g., interleukin 6 [IL-6] and C-reactive protein [CRP]). The results of this can be seen in Table 2.

Smoking was the most common mediator assessed in four papers (Graham et al., 2017; Taylor et al., 2009; Turiano, Hill, et al., 2012; Turiano et al., 2015). Each of these papers investigates how smoking mediates the personality–mortality relationship. Two studies (Turiano, Hill, et al., 2012; Turiano et al., 2015) found smoking to mediate the relationship between conscientiousness and mortality, with higher levels being associated with less smoking or less likelihood of being a smoker. Graham et al. (2017) found smoking partially mediated the positive association between neuroticism and mortality risk.

**Figure 3:**
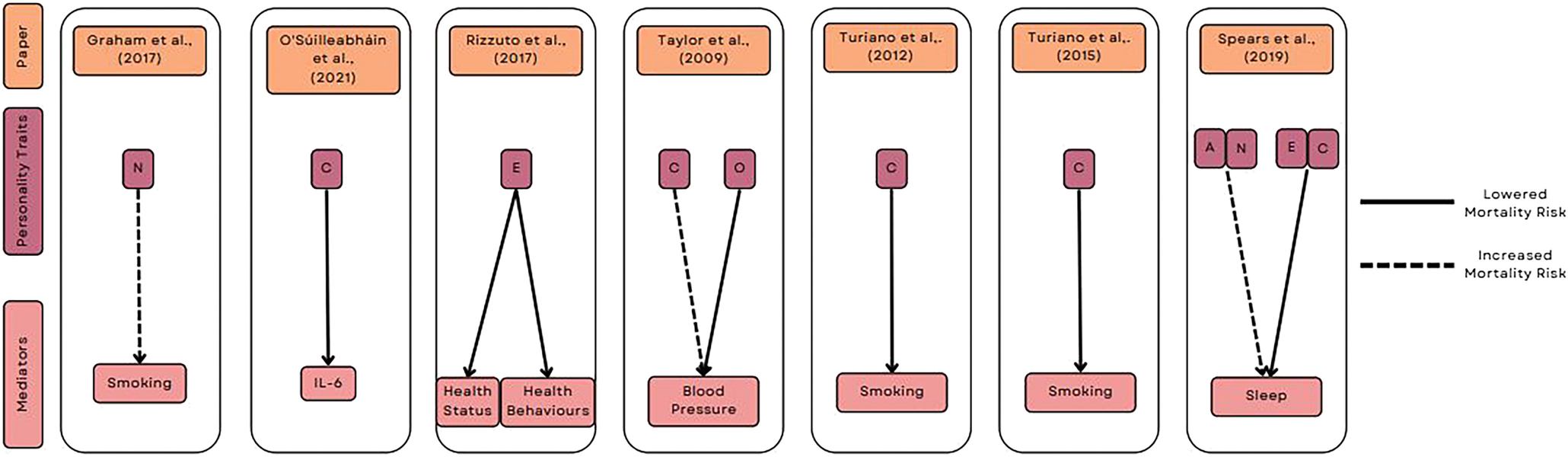
Graphical Summary of Significant Findings for Indirect Pathways Linking Personality Traits and Mortality Risk *Note.* Personality traits have been abbreviated to their initials. IL-6 = interleukin 6. See the online article for the color version of this figure.

The research by Spears et al. (2019) is the only paper that examined sleep as a mediator. Specifically, the study examined sleep duration and daytime dysfunction, with both sleep constructs being measured through self-report questionnaires. Their paper identifies that lower conscientiousness predicted increased death, mediated by low/high sleep durations and higher levels of daytime dysfunction. They also identified that higher levels of agreeableness and neuroticism predicted an increased risk of death mediated by levels of daytime dysfunction. O’Súilleabháin et al. (2021) investigated physiological biomarkers of inflammation (CRP and IL-6) as specific mediators of interest. They found that IL-6 levels partly mediated the conscientiousness to mortality pathway, with higher conscientiousness being associated with lower IL-6 levels and reduced mortality risk. Rizzuto et al.’s (2017) paper also includes physiological factors in a composite measure of health indicators, including CRP, chronic conditions, BMI, and disability status and found lifestyle and health status to mediate the relationship between extraversion and mortality. A graphical summary of significant mediation effects identified in each paper can be seen in Figure 3.

## Discussion

The present study is the first to provide a comprehensive review of the growing area of research investigating the pathways through which personality influences all-cause mortality risk. Our review identified seven longitudinal studies examining a range of mediator variables. These variables included health behaviors such as smoking, sleep, alcohol use, and biometric measurements, including waist circumference and inflammation biomarkers.

Smoking was a consistent mediator of the personality–mortality association. This aligns with prior research finding conscientious individuals have lower rates of smoking while higher levels of neuroticism are associated with higher levels of smoking (Malouff et al., 2006; Pluess & Bartley, 2015; Turiano, Hill, et al., 2012). Individuals high in neuroticism tend to experience negative emotions such as anxiety and stress more frequently (Lahey, 2009; Schneider, 2004), which can increase the likelihood of smoking as a coping behavior (Perkins & Grobe, 1992). Conscientious individuals tend to be more self-disciplined and careful, reducing the likelihood of engaging in high-risk or unhealthy behavior such as smoking (Terracciano & Costa, 2004). It is, however, surprising that extraversion was not found to increase rates of smoking, as extraversion is associated with more risk-taking behaviors in the literature (Hakulinen et al., 2015). When comparing the two papers by Turiano et al. (2015) and Turiano, Hill, et al. (2012) the potential for a high level of variance between cohorts is most apparent. Both find that smoking is a mediating factor in the relation between conscientiousness and a lower associated risk of mortality. However, direct comparisons are difficult due to reporting different types of hazard/odds ratios between papers. The differences in the impact of smoking are also clear with current smoking status’s direct impact on mortality varying between cohorts HR = 3.44 [2.74, 4.33] (Turiano et al., 2015) and HR = 1.43 [1.04, 1.98] (Turiano, Hill, et al., 2012). Graham et al.’s (2017) paper has a much larger sample size than other papers and includes participants from a wider range of demographic backgrounds due to combining multiple cohort studies in their analysis, which can give us more confidence in their findings and allowed them to conduct analysis on the homogeneity of results across the 15 studies in their coordinated analysis. Identifying that the direction of results across cohorts was largely similar but emphasized the amount of variance between cohorts. They also identified differences across cohorts finding that the effect of smoking as a mediator on mortality in the United States was twice as large as the effect size found in European cohorts (HR = 1.90 vs. HR = 1.40).

Sleep is explored as a mediator in one study (Spears et al., 2019) but is not included in any others despite having a well-researched association with health outcomes including cardiovascular disease (Cappuccio et al., 2011) and neurodegenerative dementias (Bubu et al., 2017). Spears et al. (2019) identified sleep as mediating all personality traits other than openness Specifically, lower conscientiousness was associated with increased death risk indirectly via duration of sleep and levels of daytime dysfunction. Higher levels of neuroticism and agreeableness also increased mortality risk through mediation effects of sleep duration and increased levels of daytime dysfunction and lower levels of extraversion were associated with increased mortality risk via indirect effects of higher daytime dysfunction. A small range of physiological biomarkers were investigated. O’Súilleabháin et al. (2021) investigated IL-6 and CRP, while Rizzuto et al. (2017) assessed CRP as part of a wider composite alongside smoking and alcohol use. Physiological biomarkers are an important variable to capture in the personality–health literature. While behaviors explain one step on the causal chain between personality and health outcomes, physiological changes represent another downstream effect which impacts health and, ultimately, mortality. A recent meta-analysis (Yoneda et al., 2023) highlighted the importance of physiology by examining the associations between personality traits and allostatic load—a composite measure that reflects the physiological effects of chronic stress on individuals. It was found that higher levels of neuroticism were associated with higher levels of allostatic load while levels of openness and conscientiousness were negatively associated with levels of allostatic load (Yoneda et al., 2023). O’Súilleabháin et al. (2021) findings indicate that the immune marker IL-6 levels partly mediated the conscientiousness to mortality pathway, with higher conscientiousness being associated with reduced risk of mortality partially as a result of lower circulating levels of IL-6. They suggested that the immune relationship identified likely permeates multiple health-related processes, and that it is critical to understand underlying biological pathways (O’Súilleabháin et al., 2021).

This review also identified some differences in methodology and reporting of results. The major difference in methodological approach is which personality traits papers include in their SEM analysis. All of the studies within this review also looked at direct associations between personality traits and mortality alongside SEM-based mediation analysis, but their approach to including traits varied. Several papers (Rizzuto et al., 2017; Taylor et al., 2009; Turiano, Hill, et al., 2012; Turiano et al., 2015) conducted mediation analysis only on personality traits which had a significant association with mortality in the initial regression models, dropping the nonsignificant personality traits from the SEM. However, as outlined in Zhao et al. (2010), this can obscure indirect effects of interest which are present even in the absence of direct effects. The remaining papers (Graham et al., 2017; O’Súilleabháin et al., 2021; Spears et al., 2019) included all personality traits in their SEM. The impact of this can be most clearly seen when Spears et al. (2019) initially report no direct association between agreeableness and mortality, but their SEM analysis finds that sleep significantly mediates the relation between agreeableness and mortality. This is an example of the information that Zhao et al. (2010) outlined could be missed by limiting which traits are included in mediation analysis to only those that show direct effects. Reporting of results also varied across papers, such as the many relevant statistics to report with SEM analyses (e.g., direct, indirect, total effects). Papers varied in which of these were reported, with Spears et al. (2019) reporting each alongside confidence intervals and significance values. This is in contrast to Taylor et al.’s (2009) paper which does not report indirect effects, just direct effects and individual paths effects. Rizzuto et al.’s (2017) paper also reports a mixture of data with total and direct effects but no reporting of indirect effects.

When reflecting on previous theoretical and process models (e.g., Friedman & Kern, 2014; Turiano et al., 2021), we can see multiple hypothesized pathways do appear in the empirical literature. Consistent with the emphasized importance of health behaviors, smoking behavior has been suggested as a mediating pathway within the empirical literature (Graham et al., 2017; Turiano, Hill, et al., 2012; Turiano et al., 2015). In relation to physiological function, O’Súilleabháin et al. (2021) provide important support for the examination of more than one mediator at one time. A notion consistent with Turiano et al.’s (2021) Personality Health Process Model. Specifically, O’Súilleabháin et al. (2021) identified that IL-6 may be of particular importance to future mortality risk in the context of personality. Similarly to smoking, this further suggests a very broad range of pathways linking personality and mortality risk. Other broader determinants like social function which is present in several theoretical models (e.g., Friedman & Kern, 2014; Turiano et al., 2021) has been included, although as part of a composite measure in Rizzuto et al.’s (2017) research. Nevertheless, a very broad range of actual pathways are yet to be examined. When we look at the level of complexity of the theoretical models and the multifaceted way in which personality traits can influence the risk of mortality, it is evident that meticulous and consistent presentation of findings across publications are critically important. This practice is indispensable for developing a more comprehensive understanding of how personality traits influence mortality outcomes, and through which variables the impact occurs. This review has identified disparities in reporting within the existing literature and pinpointed specific domains where enhancements in statistical and methodological methodologies are warranted for forthcoming research in the area.

### Strengths and Weaknesses

There are several strengths of the present review. We had clearly defined inclusion criteria and a detailed and comprehensive search strategy (see the online supplemental materials) and followed strict validated guidelines set out in PRISMA (Page et al., 2021). The review also focused on research utilizing modern statistical approaches to identify methodological limitations at the early stages of a growing area of personality research. The use of risk of bias screening of all papers included in the research was a strength of the study. However, the tools available for risk of bias in cohort studies (Ma et al., 2020) were not an ideal fit for the research reviewed in the present study as they focus on assessing intervention-based trials across cohorts. The present pool of research is very small. This is somewhat reflective of the challenges associated with access to appropriate longitudinal data to approximate causal processes, as well as the need for more complex statistical modeling. Thus, there are still many unexplored variables that are likely mediators of the personality–health association (e.g., medication adherence, healthcare seeking, stress). This means that although the present review is comprehensive in terms of summarizing currently available research, it is missing many key variables and crucially lacks replication of the mediators that are identified. Future research would not only benefit from testing additional key mediators, but also use meta-analytic techniques to test the robustness of effects once more studies have been published. At present, this significantly limits the conclusions that can be drawn from the literature.

## Conclusion

The impact of personality traits on mortality outcomes is a well-established phenomenon. Research exploring the pathways through which mortality risk is influenced indicates a clear direction for further research. The present review identifies various mediating pathways being implicated, clearly identifying the role that mediation analysis can play in developing more comprehensive models explaining the personality–mortality relationship. This review lays the foundation for future research in the field. It accomplishes this by identifying the variables that have been investigated as potential mediators and by outlining suggestions for methodological best practices. It achieves this by critiquing the variations in approaches used in different papers to conduct mediation analysis. Currently, there is a scarcity of research in this area. Therefore, further research is needed to broaden the range of variables examined and to employ more rigorous methodological approaches. This will help in determining the pathways that exert the greatest influence on the relationship between personality and mortality. In conclusion, the findings to date clearly show that mediation-based analysis has a key role to play in developing our understanding of the complex pathways that connect personality to mortality.

## Supplementary Material

Supplementary Material

## Figures and Tables

**Table 1:** Characteristics of Included Studies

Study	Personality measure	Sample size	Age (*M ± SD*, range)	Sex (% female)	Race	Mediators	Traits in analysis	% deceased	Data Set	Country	Length of follow-up

Graham et al. (2017) ^a^	Various measures	44,094	Age data not extractable	19,698 (45%)	Not reported	Smoking	N, E, O, A, C	Variable (range: 8.6%–99.6%)	IALSA (15 data sets)	United States United Kingdom Sweden Netherlands Australia	Variable
O’Súilleabháin et al. (2021)	MIDI	957	(58.62 ± 11.50, 35–86)	536 (56%)	White 93.3% Other 6.7%	Biological factors (interleukin-6, C-reactive protein)	N, E, O, A, C	11.6% (*N* = 111)	MIDUS	United States	23 years
Rizzuto et al. (2017)	NEO-FFI	2,298	(72 ± 9.8)	1,411 (61%)	Not reported	Lifestyle behaviors, health status	N, E, O	26.5% (*N* = 608)	SNAC-K	Sweden	11 years
Spears et al. (2019)	MIDI	3,759	(47.15 ± 12.35)	2,070 (55%)	White 94.12% Other 5.88%	Sleep (quadratic sleep duration, daytime dysfunction)	N, E, O, A, C	10.7% (*N* = 403)	MIDUS	United States	20 Years
Taylor et al. (2009)	NEO-FFI	1,322	(69.5 ± 5.6)	670 (51%)	Not reported	Age, social class, smoking, blood pressure, BMI	N, E, O, A, C	30.8% (*N* = 407)	EAS	Scotland	10 years
Turiano et al. (2015)	MIDI	6,325	(46.38 ± 13, 20–75)	2,909 (46%)	White 91% Other 9%	Alcohol use, smoking, waist circumference	N, E, O, A, C	9% (*N* = 580)	MIDUS	United States	15 years
Turiano, Whiteman, et al. (2012)	Goldberg (1992) adjectival markers	1,349	(64.9 ± 7.75, 45–89)	0 F 0% (all male)	Not recorded	Smoking	C	41% (*N* = 547)	VA-NAS	United States	17 years

*Note.* MIDI = The Midlife Development Inventory; NEO-FFI = NEO Five-Factor Inventory; IAES A = Integrative Analysis of Longitudinal Studies on Aging; MIDUS = Midlife in the United States Study; SNAC-K = Swedish National Study of Aging and Care in Kungsholmen; EAS = Edinburgh Artery Study; VA-NAS = Veterans Affairs Normative Aging Study; BMI = body mass index; N = neuroticism; E = extraversión; O = openness; A = agreeableness; C = conscientiousness.

a Graham et al.’s (2017) paper analyzed many different cohorts some information could not be collated for Table 2, demographic information by cohort detailed in the original paper.

**Table 2:** Summary of Key Findings Direct and Indirect Effects

Study	Mediator	Trait	Direct effect HR/estimate [CI]	Indirect effect HR/estimate [CI]

Spears et al. (2019)	Sleep duration	C	0.92 [0.85, 1.00]*	−0.01 [−0.02, −0.00]*
		A	0.97 [0.88, 1.06]	0.01 [0.00, 0.02]*
		N	1.01 [0.93, 1.10]	0.02 [0.01, 0.03]**
		O	1.12 [1.02, 1.23]*	0.00 [−0.01, 0.01]
		E	0.95 [0.86, 1.05]	−0.00 [−0.01, 0.01]
	Daytime dysfunction	C	0.93 [0.87, 1.00]	−0.01 [−0.02, −0.00]**
		A	0.96 [0.88, 1.04]	0.02 [0.01, 0.03]**
		N	1.00 [0.92, 1.07]	0.04 [0.02, 0.07]***
		O	1.10 [1.02, 1.19]*	−0.00 [−0.01, 0.00]
		E	0.96 [0.88, 1.05]	0.02 [−0.03, −0.01]**
O’Súilleabháin et al. (2021)	IL-6	C	0.833 [0.66, 1.00]	−0.039 [−0.07, −0.01]*
		N	1.133 [0.87, 1.39]	−0.028 [−0.06, 0.01]
	CRP	C	0.83 [0.66, 1.00]	0.003 [−0.01, 0.02]
		N	1.13 [0.87, 1.39]	0.002 [−0.01, 0.01]
Rizzuto et al. (2017)	Lifestyle behaviors	E	^a^	^a^
	Health status	E	^a^	^a^
Taylor et al. (2009)	Blood pressure	O	^a^	^a^
		C	^a^	^a^
Turiano et al. (2015)	Smoker	C	1.07 [0.98, 1.15]	0.12 [0.04, 0.20]**
	Waist circumference	C	1.07 [0.98, 1.15]	0.02 [0.01, 0.03]***
	Alcohol use	C	1.07 [0.98, 1.15]	0.04 [0.01, 0.09]*
Graham et al. (2017)	Current smoker	O	1.05 [0.90, 1.14]	1.02 [0.99, 1.06]
		C	0.99 [0.97, 1.01]	0.99 [0.98, 1.00]
		E	1.04 [1.02, 1.07]*	1.01 [0.99, 1.02]
		A	0.98 [0.95, 1.02]	1.01 [0.98, 1.03]
		N	1.04 [1.02, 1.06]*	1.02 [1.00, 1.03]*
Turiano, Whiteman, et al. (2012)	Current smoker	C	0.92 [0.85, 1.01]	0.91 [0.82, 0.96]

*Note.* HR = hazard ratio; CI = confidence interval; IL-6 = interleukin 6; CRP = C-reactive protein; N = neuroticism; E = extraversion; O = openness; A = agreeableness; C = conscientiousness.

a Direct/indirect effect not reported numerically.

* *p* < .05.

** *p* < .01.

*** *p* < .001.

## Public Significance Statement

This review found that smoking, inflammation biomarkers, blood pressure, and sleep have been identified as possible mechanisms explaining why some personality traits are related to future risk of death. In highlighting the importance of identifying underlying pathways for future healthcare practice, this review also outlines several avenues and considerations for future research.
