# Supplementary material for: Personality Traits and Mediating Pathways to Mortality Risk: A Systematic Review
Source: Health Psychol. Author manuscript; Available in PMC 2025 Apr 9. (PMC11979782; doi:10.1037/hea0001335)
Supplement: Supplementary Material [file NIHMS2061496-supplement-Supplementary_Material.pdf]

Supplemental Materials: Search Terms search 27/01/23

Pubmed - 127

("Personality"[All Fields] OR "Neuroticism"[Title/Abstract] OR "extraversion"[Title/Abstract] OR "extroversion"[Title/Abstract] OR "agreeableness"[Title/Abstract] OR "openness"[Title/Abstract] OR "conscientiousness"[Title/Abstract]) AND ("Big 5"[All Fields] OR "5-FACTOR MODEL"[All Fields] OR "Big Five personality traits"[All Fields] OR "Big Five"[All Fields] OR "five-factor model"[All Fields] OR "Big Five Inventory"[All Fields] OR "5-FACTOR MODEL"[All Fields] OR "NEO Personality Inventory"[All Fields] OR "NEO PI"[All Fields] OR "EPI-Q"[All Fields] OR "Eysenck Personality Inventory"[All Fields] OR "International Personality Item Pool"[All Fields] OR "The Ten-Item Personality Inventory"[All Fields]) AND ("Mortality"[Title/Abstract] OR "Death"[Title/Abstract] OR "longevity"[Title/Abstract])

Web of science - 177

(ALL=(Personality) OR AB=(Neuroticism) OR AB=(extraversion) OR AB=(extroversion) OR AB=(agreeableness) OR AB=(openness) OR AB=(conscientiousness)) AND (ALL=("Big 5") OR ALL=("5-FACTOR MODEL") OR ALL=("Big Five personality traits") OR ALL=("five-factor model") OR ALL=("Big Five Inventory") OR ALL=("NEO Personality Inventory”) OR ALL=("NEO PI”) OR ALL=("EPI-Q") OR ALL=("Eysenck Personality Inventory") OR ALL=("International Personality Item Pool") OR ALL=("The Ten-Item Personality Inventory")) AND (AB=("Mortality") OR AB=(Death) OR AB=("longevity"))

CINAHL, Psychinfo, Psycharticles (searched through EBSCO) – 307

(Personality OR AB (Neuroticism) OR AB (extraversion) OR AB (extroversion) OR AB (agreeableness) OR AB (openness) OR AB (conscientiousness)) AND (“Big 5” OR "5-FACTOR MODEL" OR "Big Five personality traits" OR "five-factor model" OR "Big Five Inventory" OR "NEO Personality Inventory" OR "NEO PI" OR "EPI-Q" OR "Eysenck Personality Inventory" OR "International Personality Item Pool" OR "The Ten-Item Personality Inventory") AND (AB ("Mortality") OR AB (“Death”) OR AB (“longevity”))

<u>Combined searched (PUBMED, Web of science, CINAHL, PsychInfo, PsychArticles) with duplicates removed = 411</u>

CASP
Critical Appraisal Skills Programme

www.casp-uk.net
info@casp-uk.net
Summertown Pavilion, Middle Way Oxford OX2 7LG

CASP Checklist: 12 questions to help you make sense of a Cohort Study

How to use this appraisal tool: Three broad issues need to be considered when appraising a cohort study:

- Are the results of the study valid? (Section A)
- What are the results? (Section B)
- Will the results help locally? (Section C)

The 12 questions on the following pages are designed to help you think about these issues systematically. The first two questions are screening questions and can be answered quickly. If the answer to both is "yes", it is worth proceeding with the remaining questions. There is some degree of overlap between the questions, you are asked to record a "yes", "no" or "can't tell" to most of the questions. A number of italicised prompts are given after each question. These are designed to remind you why the question is important. Record your reasons for your answers in the spaces provided.

About: These checklists were designed to be used as educational pedagogic tools, as part of a workshop setting, therefore we do not suggest a scoring system. The core CASP checklists (randomised controlled trial & systematic review) were based on JAMA 'Users' guides to the medical literature 1994 (adapted from Guyatt GH, Sackett DL, and Cook DJ), and piloted with health care practitioners.

For each new checklist, a group of experts were assembled to develop and pilot the checklist and the workshop format with which it would be used. Over the years overall adjustments have been made to the format, but a recent survey of checklist users reiterated that the basic format continues to be useful and appropriate.

Referencing: we recommend using the Harvard style citation, i.e.: *Critical Appraisal Skills Programme (2018). CASP (insert name of checklist i.e. Cohort Study) Checklist. [online] Available at: URL. Accessed: Date Accessed.*

©CASP this work is licensed under the Creative Commons Attribution – Non-Commercial-Share A like. To view a copy of this license, visit http://creativecommons.org/licenses/by-nc-sa/3.0/ www.casp-uk.net

CASP
Critical Appraisal
Skills Programme

Paper for appraisal and reference: Smoking mediates the effect of conscientiousness on mor

Section A: Are the results of the study valid?

1. Did the study address a clearly focused issue?

Yes ✔
Can't Tell ☐
No ☐

HINT: A question can be 'focused' in terms of
- the population studied
- the risk factors studied
- is it clear whether the study tried to detect a beneficial or harmful effect
- the outcomes considered

Comments: This paper focuses directly on the question at hand and provides evidence towards smoking being a mediator of the C - Mortality relationship.

2. Was the cohort recruited in an acceptable way?

Yes ✔
Can't Tell ☐
No ☐

HINT: Look for selection bias which might compromise the generalisability of the findings:
- was the cohort representative of a defined population
- was there something special about the cohort
- was everybody included who should have been

Comments: Cohort comes from the The Veterans Affairs Normative Aging Study, a large cohort study primarily gathered to do research on aging. This paper uses a subsection of 1349 participants who completed personality measures. This cohort is acceptable in terms of size and recruitment.

Note that the cohort is only male and from a military veteran population.

Is it worth continuing?

3. Was the exposure accurately measured to minimise bias?

Yes ✔
Can't Tell ☐
No ☐

HINT: Look for measurement or classification bias:
- did they use subjective or objective measurements
- do the measurements truly reflect what you want them to (have they been validated)
- were all the subjects classified into exposure groups using the same procedure

Comments: due to the nature of the studies there was not an exposure. Answering this question I will refer to measurement of personality and other variables of note:

Personality: Measured using Goldberg adjetive marker big 5 test

education: measured as a continous variable with detailed input ranging from primary school to college degree
Smoking was recorded and split into 3 catagories: Former, current and non smoker

A

4. Was the outcome accurately measured to minimise bias?

Yes ✔
Can't Tell ☐
No ☐

HINT: Look for measurement or classification bias:
- did they use subjective or objective measurements
- do the measurements truly reflect what you want them to (have they been validated)
- has a reliable system been established for detecting all the cases (for measuring disease occurrence)
- were the measurement methods similar in the different groups
- were the subjects and/or the outcome assessor blinded to exposure (does this matter)

Comments: Periodic contacting is utilized to get information about mortality and then death certificates are collected for conformation of date.

5. (a) Have the authors identified all important confounding factors?

Yes ✔
Can't Tell ☐
No ☐

HINT:
- list the ones you think might be important, and ones the author missed

Comments: Age and education are accounted for. Gender is not applicable.

Race is not included in the study as a variable.

5. (b) Have they taken account of the confounding factors in the design and/or analysis?

Yes ✔
Can't Tell ☐
No ☐

HINT:
- look for restriction in design, and techniques e.g. modelling, stratified-, regression-, or sensitivity analysis to correct, control or adjust for confounding factors

Comments:

6. (a) Was the follow up of subjects complete enough?

Yes ✔
Can't Tell ☐
No ☐

HINT: Consider
- the good or bad effects should have had long enough to reveal themselves
- the persons that are lost to follow-up may have different outcomes than those available for assessment
- in an open or dynamic cohort, was there anything special about the outcome of the people leaving, or the exposure of the people entering the cohort

6. (b) Was the follow up of subjects long enough?

Yes ✔
Can't Tell ☐
No ☐

CASP
Critical Appraisal
Skills Programme

Comments: 17 year follow up with 547 (46%) of cohort having died.

Section B: What are the results?

7. What are the results of this study?

HINT: Consider
- what are the bottom line results
- have they reported the rate or the proportion between the exposed/unexposed, the ratio/rate difference
- how strong is the association between exposure and outcome (RR)
- what is the absolute risk reduction (ARR)

Comments: proportional hazards analyses confirmed that conscientiousness predicted mortality risk in an unadjusted model (HR = 0.84; CI = 0.77–0.91; p < .001). Adjusting for age, education, and self-rated health reduced the direct effect of conscientiousness but it was still significant (HR = 0.91; CI = 0.84–0.99; p < 0.05). For a standard deviation increase in conscientiousness (about 1 point) there was a 9% decreased hazard of dying.

A standard deviation increase in conscientiousness was significantly associated with an 18% decreased odds of being a current smoker (path a1) and in turn, being a current smoker was associated with a 43% increased hazard of dying (path b1) over the 18-year follow-up period

8. How precise are the results?

HINT:
- look for the range of the confidence intervals, if given

Comments: Total effects are small within the study.

Total C effect on mortality = 0.84 [0.74–0.96]
Simialrly impact of C on being a current smoker is small but sig = 0.82 [0.67-0.94]

The confidence intervals within this research are large with potentially very small effects however due to the outcome metric being mortality this is still valuable information.

CASP
Critical Appraisal
Skills Programme

9. Do you believe the results?

Yes ✔
Can't Tell ☐
No ☐

HINT: Consider
- big effect is hard to ignore
- can it be due to bias, chance or confounding
- are the design and methods of this study sufficiently flawed to make the results unreliable
- Bradford Hills criteria (e.g. time sequence, dose-response gradient, biological plausibility, consistency)

Comments: Results of this study show significant results and indicate that personality to mortality relationship is mediated by how conscientiousness impacts on smoking habits.

Section C: Will the results help locally?

10. Can the results be applied to the local population?

Yes ☐
Can't Tell ☐
No ✔

HINT: Consider whether
- a cohort study was the appropriate method to answer this question
- the subjects covered in this study could be sufficiently different from your population to cause concern
- your local setting is likely to differ much from that of the study
- you can quantify the local benefits and harms

Comments: No

11. Do the results of this study fit with other available evidence?

Yes ✔
Can't Tell ☐
No ☐

Comments: This study brings together our knowledge of the impact of personality traits on both longevity and personalities impact on certain health behaviour (smoking in this case).

CASP
Critical Appraisal
Skills Programme

12. What are the implications of this study for practice?

Yes ☐

Can't Tell ☑

No ☐

HINT: Consider
- one observational study rarely provides sufficiently robust evidence to recommend changes to clinical practice or within health policy decision making
- for certain questions, observational studies provide the only evidence
- recommendations from observational studies are always stronger when supported by other evidence

Comments: This area of research is too early for it to have impacts on clinical health practise.

CASP
Critical Appraisal
Skills Programme

www.casp-uk.net

info@casp-uk.net

Summertown Pavilion, Middle
Way Oxford OX2 7LG

CASP Checklist: 12 questions to help you make sense of a Cohort Study

How to use this appraisal tool: Three broad issues need to be considered when appraising a cohort study:

- Are the results of the study valid? (Section A)
- What are the results? (Section B)
- Will the results help locally? (Section C)

The 12 questions on the following pages are designed to help you think about these issues systematically. The first two questions are screening questions and can be answered quickly. If the answer to both is "yes", it is worth proceeding with the remaining questions. There is some degree of overlap between the questions, you are asked to record a "yes", "no" or "can't tell" to most of the questions. A number of italicised prompts are given after each question. These are designed to remind you why the question is important. Record your reasons for your answers in the spaces provided.

About: These checklists were designed to be used as educational pedagogic tools, as part of a workshop setting, therefore we do not suggest a scoring system. The core CASP checklists (randomised controlled trial & systematic review) were based on JAMA 'Users' guides to the medical literature 1994 (adapted from Guyatt GH, Sackett DL, and Cook DJ), and piloted with health care practitioners.

For each new checklist, a group of experts were assembled to develop and pilot the checklist and the workshop format with which it would be used. Over the years overall adjustments have been made to the format, but a recent survey of checklist users reiterated that the basic format continues to be useful and appropriate.

Referencing: we recommend using the Harvard style citation, i.e.: *Critical Appraisal Skills Programme (2018). CASP (insert name of checklist i.e. Cohort Study) Checklist. [online] Available at: URL. Accessed: Date Accessed.*

©CASP this work is licensed under the Creative Commons Attribution – Non-Commercial-Share A like. To view a copy of this license, visit http://creativecommons.org/licenses/by-nc-sa/3.0/ www.casp-uk.net

CASP
Critical Appraisal
Skills Programme

Paper for appraisal and reference: Smoking mediates the effect of conscientiousness on mor

Section A: Are the results of the study valid?

1. Did the study address a clearly focused issue?

Yes ✔
Can't Tell ☐
No ☐

HINT: A question can be 'focused' in terms of
- the population studied
- the risk factors studied
- is it clear whether the study tried to detect a beneficial or harmful effect
- the outcomes considered

Comments: This paper focuses directly on the question at hand and provides evidence towards smoking being a mediator of the C - Mortality relationship.

2. Was the cohort recruited in an acceptable way?

Yes ✔
Can't Tell ☐
No ☐

HINT: Look for selection bias which might compromise the generalisability of the findings:
- was the cohort representative of a defined population
- was there something special about the cohort
- was everybody included who should have been

Comments: Cohort comes from the The Veterans Affairs Normative Aging Study, a large cohort study primarily gathered to do research on aging. This paper uses a subsection of 1349 participants who completed personality measures. This cohort is acceptable in terms of size and recruitment.

Note that the cohort is only male and from a military veteran population.

Is it worth continuing?

CASP
Critical Appraisal
Skills Programme

3. Was the exposure accurately measured to minimise bias?

Yes	✔
Can't Tell	
No	

HINT: Look for measurement or classification bias:
- did they use subjective or objective measurements
- do the measurements truly reflect what you want them to (have they been validated)
- were all the subjects classified into exposure groups using the same procedure

Comments: due to the nature of the studies there was not an exposure. Answering this question I will refer to measurement of personality and other variables of note:

Personality: Measured using Goldberg adjetive marker big 5 test

education: measured as a continous variable with detailed input ranging from primary school to college degree
Smoking was recorded and split into 3 catagories: Former, current and non smoker

A

4. Was the outcome accurately measured to minimise bias?

Yes	✔
Can't Tell	
No	

HINT: Look for measurement or classification bias:
- did they use subjective or objective measurements
- do the measurements truly reflect what you want them to (have they been validated)
- has a reliable system been established for detecting all the cases (for measuring disease occurrence)
- were the measurement methods similar in the different groups
- were the subjects and/or the outcome assessor blinded to exposure (does this matter)

Comments: Periodic contacting is utilized to get information about mortality and then death certificates are collected for conformation of date.

CASP
Critical Appraisal
Skills Programme

5. (a) Have the authors identified all important confounding factors?

Yes ✔
Can't Tell ☐
No ☐

HINT:
- list the ones you think might be important, and ones the author missed

Comments: Age and education are accounted for. Gender is not applicable.

Race is not included in the study as a variable.

5. (b) Have they taken account of the confounding factors in the design and/or analysis?

Yes ✔
Can't Tell ☐
No ☐

HINT:
- look for restriction in design, and techniques e.g. modelling, stratified-, regression-, or sensitivity analysis to correct, control or adjust for confounding factors

Comments:

6. (a) Was the follow up of subjects complete enough?

Yes ✔
Can't Tell ☐
No ☐

HINT: Consider
- the good or bad effects should have had long enough to reveal themselves
- the persons that are lost to follow-up may have different outcomes than those available for assessment
- in an open or dynamic cohort, was there anything special about the outcome of the people leaving, or the exposure of the people entering the cohort

6. (b) Was the follow up of subjects long enough?

Yes ✔
Can't Tell ☐
No ☐

Comments: 17 year follow up with 547 (46%) of cohort having died.

Section B: What are the results?

7. What are the results of this study?

HINT: Consider
- what are the bottom line results
- have they reported the rate or the proportion between the exposed/unexposed, the ratio/rate difference
- how strong is the association between exposure and outcome (RR)
- what is the absolute risk reduction (ARR)

Comments: proportional hazards analyses confirmed that conscientiousness predicted mortality risk in an unadjusted model (HR = 0.84; CI = 0.77–0.91; p < .001). Adjusting for age, education, and self-rated health reduced the direct effect of conscientiousness but it was still significant (HR = 0.91; CI = 0.84–0.99; p < 0.05). For a standard deviation increase in conscientiousness (about 1 point) there was a 9% decreased hazard of dying.

A standard deviation increase in conscientiousness was significantly associated with an 18% decreased odds of being a current smoker (path a1) and in turn, being a current smoker was associated with a 43% increased hazard of dying (path b1) over the 18-year follow-up period

8. How precise are the results?

HINT:
- look for the range of the confidence intervals, if given

Comments: Total effects are small within the study.

Total C effect on mortality = 0.84 [0.74–0.96]
Simialrly impact of C on being a current smoker is small but sig = 0.82 [0.67-0.94]

The confidence intervals within this research are large with potentially very small effects however due to the outcome metric being mortality this is still valuable information.

9. Do you believe the results?

Yes ✔
Can't Tell ☐
No ☐

HINT: Consider
- big effect is hard to ignore
- can it be due to bias, chance or confounding
- are the design and methods of this study sufficiently flawed to make the results unreliable
- Bradford Hills criteria (e.g. time sequence, dose-response gradient, biological plausibility, consistency)

Comments: Results of this study show significant results and indicate that personality to mortality relationship is mediated by how conscientiousness impacts on smoking habits.

Section C: Will the results help locally?

10. Can the results be applied to the local population?

Yes ☐
Can't Tell ☐
No ✔

HINT: Consider whether
- a cohort study was the appropriate method to answer this question
- the subjects covered in this study could be sufficiently different from your population to cause concern
- your local setting is likely to differ much from that of the study
- you can quantify the local benefits and harms

Comments: No

11. Do the results of this study fit with other available evidence?

Yes ✔
Can't Tell ☐
No ☐

Comments: This study brings together our knowledge of the impact of personality traits on both longevity and personalities impact on certain health behaviour (smoking in this case).

12. What are the implications of this study for practice?

Yes	☐
Can't Tell	☑
No	☐

HINT: Consider
- one observational study rarely provides sufficiently robust evidence to recommend changes to clinical practice or within health policy decision making
- for certain questions, observational studies provide the only evidence
- recommendations from observational studies are always stronger when supported by other evidence

Comments: This area of research is too early for it to have impacts on clinical health practise.

CASP
Critical Appraisal Skills Programme

www.casp-uk.net
info@casp-uk.net
Summertown Pavilion, Middle Way Oxford OX2 7LG

CASP Checklist: 12 questions to help you make sense of a Cohort Study

How to use this appraisal tool: Three broad issues need to be considered when appraising a cohort study:

- Are the results of the study valid? (Section A)
- What are the results? (Section B)
- Will the results help locally? (Section C)

The 12 questions on the following pages are designed to help you think about these issues systematically. The first two questions are screening questions and can be answered quickly. If the answer to both is "yes", it is worth proceeding with the remaining questions. There is some degree of overlap between the questions, you are asked to record a "yes", "no" or "can't tell" to most of the questions. A number of italicised prompts are given after each question. These are designed to remind you why the question is important. Record your reasons for your answers in the spaces provided.

About: These checklists were designed to be used as educational pedagogic tools, as part of a workshop setting, therefore we do not suggest a scoring system. The core CASP checklists (randomised controlled trial & systematic review) were based on JAMA 'Users' guides to the medical literature 1994 (adapted from Guyatt GH, Sackett DL, and Cook DJ), and piloted with health care practitioners.

For each new checklist, a group of experts were assembled to develop and pilot the checklist and the workshop format with which it would be used. Over the years overall adjustments have been made to the format, but a recent survey of checklist users reiterated that the basic format continues to be useful and appropriate.

Referencing: we recommend using the Harvard style citation, i.e.: *Critical Appraisal Skills Programme (2018). CASP (insert name of checklist i.e. Cohort Study) Checklist. [online] Available at: URL. Accessed: Date Accessed.*

©CASP this work is licensed under the Creative Commons Attribution – Non-Commercial-Share A like. To view a copy of this license, visit http://creativecommons.org/licenses/by-nc-sa/3.0/ www.casp-uk.net

CASP
Critical Appraisal
Skills Programme

Paper for appraisal and reference: Smoking mediates the effect of conscientiousness on mor

Section A: Are the results of the study valid?

1. Did the study address a clearly focused issue?

Yes ✔
Can't Tell ☐
No ☐

HINT: A question can be 'focused' in terms of
- the population studied
- the risk factors studied
- is it clear whether the study tried to detect a beneficial or harmful effect
- the outcomes considered

Comments: This paper focuses directly on the question at hand and provides evidence towards smoking being a mediator of the C - Mortality relationship.

2. Was the cohort recruited in an acceptable way?

Yes ✔
Can't Tell ☐
No ☐

HINT: Look for selection bias which might compromise the generalisability of the findings:
- was the cohort representative of a defined population
- was there something special about the cohort
- was everybody included who should have been

Comments: Cohort comes from the The Veterans Affairs Normative Aging Study, a large cohort study primarily gathered to do research on aging. This paper uses a subsection of 1349 participants who completed personality measures. This cohort is acceptable in terms of size and recruitment.

Note that the cohort is only male and from a military veteran population.

Is it worth continuing?

Critical Appraisal Skills Programme

3. Was the exposure accurately measured to minimise bias?

Yes ✔
Can't Tell
No

HINT: Look for measurement or classification bias:

- did they use subjective or objective measurements
- do the measurements truly reflect what you want them to (have they been validated)
- were all the subjects classified into exposure groups using the same procedure

Comments: due to the nature of the studies there was not an exposure. Answering this question I will refer to measurement of personality and other variables of note:

Personality: Measured using Goldberg adjetive marker big 5 test

education: measured as a continous variable with detailed input ranging from primary school to college degree
Smoking was recorded and split into 3 catagories: Former, current and non smoker

A

4. Was the outcome accurately measured to minimise bias?

Yes ✔
Can't Tell
No

HINT: Look for measurement or classification bias:

- did they use subjective or objective measurements
- do the measurements truly reflect what you want them to (have they been validated)
- has a reliable system been established for detecting all the cases (for measuring disease occurrence)
- were the measurement methods similar in the different groups
- were the subjects and/or the outcome assessor blinded to exposure (does this matter)

Comments: Periodic contacting is utilized to get information about mortality and then death certificates are collected for conformation of date.

5. (a) Have the authors identified all important confounding factors?

Yes ✔
Can't Tell ☐
No ☐

HINT:
- list the ones you think might be important, and ones the author missed

Comments: Age and education are accounted for. Gender is not applicable.

Race is not included in the study as a variable.

5. (b) Have they taken account of the confounding factors in the design and/or analysis?

Yes ✔
Can't Tell ☐
No ☐

HINT:
- look for restriction in design, and techniques e.g. modelling, stratified-, regression-, or sensitivity analysis to correct, control or adjust for confounding factors

Comments:

6. (a) Was the follow up of subjects complete enough?

Yes ✔
Can't Tell ☐
No ☐

HINT: Consider
- the good or bad effects should have had long enough to reveal themselves
- the persons that are lost to follow-up may have different outcomes than those available for assessment
- in an open or dynamic cohort, was there anything special about the outcome of the people leaving, or the exposure of the people entering the cohort

6. (b) Was the follow up of subjects long enough?

Yes ✔
Can't Tell ☐
No ☐

Comments: 17 year follow up with 547 (46%) of cohort having died.

Section B: What are the results?

7. What are the results of this study?

HINT: Consider
- what are the bottom line results
- have they reported the rate or the proportion between the exposed/unexposed, the ratio/rate difference
- how strong is the association between exposure and outcome (RR)
- what is the absolute risk reduction (ARR)

Comments: proportional hazards analyses confirmed that conscientiousness predicted mortality risk in an unadjusted model (HR = 0.84; CI = 0.77–0.91; p < .001). Adjusting for age, education, and self-rated health reduced the direct effect of conscientiousness but it was still significant (HR = 0.91; CI = 0.84–0.99; p < 0.05). For a standard deviation increase in conscientiousness (about 1 point) there was a 9% decreased hazard of dying.

A standard deviation increase in conscientiousness was significantly associated with an 18% decreased odds of being a current smoker (path a1) and in turn, being a current smoker was associated with a 43% increased hazard of dying (path b1) over the 18-year follow-up period

8. How precise are the results?

HINT:
- look for the range of the confidence intervals, if given

Comments: Total effects are small within the study.

Total C effect on mortality = 0.84 [0.74–0.96]
Simialrly impact of C on being a current smoker is small but sig = 0.82 [0.67-0.94]

The confidence intervals within this research are large with potentially very small effects however due to the outcome metric being mortality this is still valuable information.

9. Do you believe the results?

Yes ☑
Can't Tell ☐
No ☐

HINT: Consider
- big effect is hard to ignore
- can it be due to bias, chance or confounding
- are the design and methods of this study sufficiently flawed to make the results unreliable
- Bradford Hills criteria (e.g. time sequence, dose-response gradient, biological plausibility, consistency)

Comments: Results of this study show significant results and indicate that personality to mortality relationship is mediated by how conscientiousness impacts on smoking habits.

Section C: Will the results help locally?

10. Can the results be applied to the local population?

Yes ☐
Can't Tell ☐
No ☑

HINT: Consider whether
- a cohort study was the appropriate method to answer this question
- the subjects covered in this study could be sufficiently different from your population to cause concern
- your local setting is likely to differ much from that of the study
- you can quantify the local benefits and harms

Comments: No

11. Do the results of this study fit with other available evidence?

Yes ☑
Can't Tell ☐
No ☐

Comments: This study brings together our knowledge of the impact of personality traits on both longevity and personalities impact on certain health behaviour (smoking in this case).

12. What are the implications of this study for practice?

Yes	☐
Can't Tell	☑
No	☐

HINT: Consider
- one observational study rarely provides sufficiently robust evidence to recommend changes to clinical practice or within health policy decision making
- for certain questions, observational studies provide the only evidence
- recommendations from observational studies are always stronger when supported by other evidence

Comments: This area of research is too early for it to have impacts on clinical health practise.

CASP
Critical Appraisal Skills Programme

www.casp-uk.net
info@casp-uk.net
Summertown Pavilion, Middle Way Oxford OX2 7LG

CASP Checklist: 12 questions to help you make sense of a Cohort Study

How to use this appraisal tool: Three broad issues need to be considered when appraising a cohort study:

- Are the results of the study valid? (Section A)
- What are the results? (Section B)
- Will the results help locally? (Section C)

The 12 questions on the following pages are designed to help you think about these issues systematically. The first two questions are screening questions and can be answered quickly. If the answer to both is "yes", it is worth proceeding with the remaining questions. There is some degree of overlap between the questions, you are asked to record a "yes", "no" or "can't tell" to most of the questions. A number of italicised prompts are given after each question. These are designed to remind you why the question is important. Record your reasons for your answers in the spaces provided.

About: These checklists were designed to be used as educational pedagogic tools, as part of a workshop setting, therefore we do not suggest a scoring system. The core CASP checklists (randomised controlled trial & systematic review) were based on JAMA 'Users' guides to the medical literature 1994 (adapted from Guyatt GH, Sackett DL, and Cook DJ), and piloted with health care practitioners.

For each new checklist, a group of experts were assembled to develop and pilot the checklist and the workshop format with which it would be used. Over the years overall adjustments have been made to the format, but a recent survey of checklist users reiterated that the basic format continues to be useful and appropriate.

Referencing: we recommend using the Harvard style citation, i.e.: *Critical Appraisal Skills Programme (2018). CASP (insert name of checklist i.e. Cohort Study) Checklist. [online] Available at: URL. Accessed: Date Accessed.*

©CASP this work is licensed under the Creative Commons Attribution – Non-Commercial-Share A like. To view a copy of this license, visit http://creativecommons.org/licenses/by-nc-sa/3.0/ www.casp-uk.net

CASP
Critical Appraisal
Skills Programme

Paper for appraisal and reference: Smoking mediates the effect of conscientiousness on mor

Section A: Are the results of the study valid?

1. Did the study address a clearly focused issue?

Yes ✔
Can't Tell ☐
No ☐

HINT: A question can be 'focused' in terms of
- the population studied
- the risk factors studied
- is it clear whether the study tried to detect a beneficial or harmful effect
- the outcomes considered

Comments: This paper focuses directly on the question at hand and provides evidence towards smoking being a mediator of the C - Mortality relationship.

2. Was the cohort recruited in an acceptable way?

Yes ✔
Can't Tell ☐
No ☐

HINT: Look for selection bias which might compromise the generalisability of the findings:
- was the cohort representative of a defined population
- was there something special about the cohort
- was everybody included who should have been

Comments: Cohort comes from the The Veterans Affairs Normative Aging Study, a large cohort study primarily gathered to do research on aging. This paper uses a subsection of 1349 participants who completed personality measures. This cohort is acceptable in terms of size and recruitment.

Note that the cohort is only male and from a military veteran population.

Is it worth continuing?

CASP
Critical Appraisal
Skills Programme

3. Was the exposure accurately measured to minimise bias?

Yes ☑
Can't Tell ☐
No ☐

HINT: Look for measurement or classification bias:
- did they use subjective or objective measurements
- do the measurements truly reflect what you want them to (have they been validated)
- were all the subjects classified into exposure groups using the same procedure

Comments: due to the nature of the studies there was not an exposure. Answering this question I will refer to measurement of personality and other variables of note:

Personality: Measured using Goldberg adjetive marker big 5 test

education: measured as a continous variable with detailed input ranging from primary school to college degree
Smoking was recorded and split into 3 catagories: Former, current and non smoker

A

4. Was the outcome accurately measured to minimise bias?

Yes ☑
Can't Tell ☐
No ☐

HINT: Look for measurement or classification bias:
- did they use subjective or objective measurements
- do the measurements truly reflect what you want them to (have they been validated)
- has a reliable system been established for detecting all the cases (for measuring disease occurrence)
- were the measurement methods similar in the different groups
- were the subjects and/or the outcome assessor blinded to exposure (does this matter)

Comments: Periodic contacting is utilized to get information about mortality and then death certificates are collected for conformation of date.

5. (a) Have the authors identified all important confounding factors?

Yes ✔
Can't Tell
No

HINT:
- list the ones you think might be important, and ones the author missed

Comments: Age and education are accounted for. Gender is not applicable.

Race is not included in the study as a variable.

5. (b) Have they taken account of the confounding factors in the design and/or analysis?

Yes ✔
Can't Tell
No

HINT:
- look for restriction in design, and techniques e.g. modelling, stratified-, regression-, or sensitivity analysis to correct, control or adjust for confounding factors

Comments:

6. (a) Was the follow up of subjects complete enough?

Yes ✔
Can't Tell
No

HINT: Consider
- the good or bad effects should have had long enough to reveal themselves
- the persons that are lost to follow-up may have different outcomes than those available for assessment
- in an open or dynamic cohort, was there anything special about the outcome of the people leaving, or the exposure of the people entering the cohort

6. (b) Was the follow up of subjects long enough?

Yes ✔
Can't Tell
No

Comments: 17 year follow up with 547 (46%) of cohort having died.

Section B: What are the results?

7. What are the results of this study?

HINT: Consider
- what are the bottom line results
- have they reported the rate or the proportion between the exposed/unexposed, the ratio/rate difference
- how strong is the association between exposure and outcome (RR)
- what is the absolute risk reduction (ARR)

Comments: proportional hazards analyses confirmed that conscientiousness predicted mortality risk in an unadjusted model (HR = 0.84; CI = 0.77–0.91; p < .001). Adjusting for age, education, and self-rated health reduced the direct effect of conscientiousness but it was still significant (HR = 0.91; CI = 0.84–0.99; p < 0.05). For a standard deviation increase in conscientiousness (about 1 point) there was a 9% decreased hazard of dying.

A standard deviation increase in conscientiousness was significantly associated with an 18% decreased odds of being a current smoker (path a1) and in turn, being a current smoker was associated with a 43% increased hazard of dying (path b1) over the 18-year follow-up period

8. How precise are the results?

HINT:
- look for the range of the confidence intervals, if given

Comments: Total effects are small within the study.

Total C effect on mortality = 0.84 [0.74–0.96]
Simialrly impact of C on being a current smoker is small but sig = 0.82 [0.67-0.94]

The confidence intervals within this research are large with potentially very small effects however due to the outcome metric being mortality this is still valuable information.

9. Do you believe the results?

Yes	✔
Can't Tell	
No	

HINT: Consider
- big effect is hard to ignore
- can it be due to bias, chance or confounding
- are the design and methods of this study sufficiently flawed to make the results unreliable
- Bradford Hills criteria (e.g. time sequence, dose-response gradient, biological plausibility, consistency)

Comments: Results of this study show significant results and indicate that personality to mortality relationship is mediated by how conscientiousness impacts on smoking habits.

Section C: Will the results help locally?

10. Can the results be applied to the local population?

Yes	
Can't Tell	
No	✔

HINT: Consider whether
- a cohort study was the appropriate method to answer this question
- the subjects covered in this study could be sufficiently different from your population to cause concern
- your local setting is likely to differ much from that of the study
- you can quantify the local benefits and harms

Comments: No

11. Do the results of this study fit with other available evidence?

Yes	✔
Can't Tell	
No	

Comments: This study brings together our knowledge of the impact of personality traits on both longevity and personalities impact on certain health behaviour (smoking in this case).

12. What are the implications of this study for practice?

Yes ☐
Can't Tell ☑
No ☐

HINT: Consider
- one observational study rarely provides sufficiently robust evidence to recommend changes to clinical practice or within health policy decision making
- for certain questions, observational studies provide the only evidence
- recommendations from observational studies are always stronger when supported by other evidence

Comments: This area of research is too early for it to have impacts on clinical health practise.

www.casp-uk.net
info@casp-uk.net
Summertown Pavilion, Middle Way Oxford OX2 7LG

CASP Checklist: 12 questions to help you make sense of a Cohort Study

How to use this appraisal tool: Three broad issues need to be considered when appraising a cohort study:

- Are the results of the study valid? (Section A)
- What are the results? (Section B)
- Will the results help locally? (Section C)

The 12 questions on the following pages are designed to help you think about these issues systematically. The first two questions are screening questions and can be answered quickly. If the answer to both is "yes", it is worth proceeding with the remaining questions. There is some degree of overlap between the questions, you are asked to record a "yes", "no" or "can't tell" to most of the questions. A number of italicised prompts are given after each question. These are designed to remind you why the question is important. Record your reasons for your answers in the spaces provided.

About: These checklists were designed to be used as educational pedagogic tools, as part of a workshop setting, therefore we do not suggest a scoring system. The core CASP checklists (randomised controlled trial & systematic review) were based on JAMA 'Users' guides to the medical literature 1994 (adapted from Guyatt GH, Sackett DL, and Cook DJ), and piloted with health care practitioners.

For each new checklist, a group of experts were assembled to develop and pilot the checklist and the workshop format with which it would be used. Over the years overall adjustments have been made to the format, but a recent survey of checklist users reiterated that the basic format continues to be useful and appropriate.

Referencing: we recommend using the Harvard style citation, i.e.: *Critical Appraisal Skills Programme (2018). CASP (insert name of checklist i.e. Cohort Study) Checklist. [online] Available at: URL. Accessed: Date Accessed.*

©CASP this work is licensed under the Creative Commons Attribution – Non-Commercial-Share A like. To view a copy of this license, visit http://creativecommons.org/licenses/by-nc-sa/3.0/ www.casp-uk.net

CASP
Critical Appraisal
Skills Programme

Paper for appraisal and reference: Smoking mediates the effect of conscientiousness on mor

Section A: Are the results of the study valid?

1. Did the study address a clearly focused issue?

Yes	✔
Can't Tell	
No	

HINT: A question can be 'focused' in terms of
- the population studied
- the risk factors studied
- is it clear whether the study tried to detect a beneficial or harmful effect
- the outcomes considered

Comments: This paper focuses directly on the question at hand and provides evidence towards smoking being a mediator of the C - Mortality relationship.

2. Was the cohort recruited in an acceptable way?

Yes	✔
Can't Tell	
No	

HINT: Look for selection bias which might compromise the generalisability of the findings:
- was the cohort representative of a defined population
- was there something special about the cohort
- was everybody included who should have been

Comments: Cohort comes from the The Veterans Affairs Normative Aging Study, a large cohort study primarily gathered to do research on aging. This paper uses a subsection of 1349 participants who completed personality measures. This cohort is acceptable in terms of size and recruitment.

Note that the cohort is only male and from a military veteran population.

Is it worth continuing?

Critical Appraisal Skills Programme

3. Was the exposure accurately measured to minimise bias?

Yes ☑ Can't Tell ☐ No ☐

HINT: Look for measurement or classification bias:
- did they use subjective or objective measurements
- do the measurements truly reflect what you want them to (have they been validated)
- were all the subjects classified into exposure groups using the same procedure

Comments: due to the nature of the studies there was not an exposure. Answering this question I will refer to measurement of personality and other variables of note:

Personality: Measured using Goldberg adjetive marker big 5 test

education: measured as a continous variable with detailed input ranging from primary school to college degree
Smoking was recorded and split into 3 catagories: Former, current and non smoker

A

4. Was the outcome accurately measured to minimise bias?

Yes ☑ Can't Tell ☐ No ☐

HINT: Look for measurement or classification bias:
- did they use subjective or objective measurements
- do the measurements truly reflect what you want them to (have they been validated)
- has a reliable system been established for detecting all the cases (for measuring disease occurrence)
- were the measurement methods similar in the different groups
- were the subjects and/or the outcome assessor blinded to exposure (does this matter)

Comments: Periodic contacting is utilized to get information about mortality and then death certificates are collected for conformation of date.

CASP
Critical Appraisal
Skills Programme

5. (a) Have the authors identified all important confounding factors?

Yes ✔
Can't Tell ☐
No ☐

HINT:
- list the ones you think might be important, and ones the author missed

Comments: Age and education are accounted for. Gender is not applicable.

Race is not included in the study as a variable.

5. (b) Have they taken account of the confounding factors in the design and/or analysis?

Yes ✔
Can't Tell ☐
No ☐

HINT:
- look for restriction in design, and techniques e.g. modelling, stratified-, regression-, or sensitivity analysis to correct, control or adjust for confounding factors

Comments:

6. (a) Was the follow up of subjects complete enough?

Yes ✔
Can't Tell ☐
No ☐

HINT: Consider
- the good or bad effects should have had long enough to reveal themselves
- the persons that are lost to follow-up may have different outcomes than those available for assessment
- in an open or dynamic cohort, was there anything special about the outcome of the people leaving, or the exposure of the people entering the cohort

6. (b) Was the follow up of subjects long enough?

Yes ✔
Can't Tell ☐
No ☐

Comments: 17 year follow up with 547 (46%) of cohort having died.

Section B: What are the results?

7. What are the results of this study?

HINT: Consider
- what are the bottom line results
- have they reported the rate or the proportion between the exposed/unexposed, the ratio/rate difference
- how strong is the association between exposure and outcome (RR)
- what is the absolute risk reduction (ARR)

Comments: proportional hazards analyses confirmed that conscientiousness predicted mortality risk in an unadjusted model (HR = 0.84; CI = 0.77–0.91; p < .001). Adjusting for age, education, and self-rated health reduced the direct effect of conscientiousness but it was still significant (HR = 0.91; CI = 0.84–0.99; p < 0.05). For a standard deviation increase in conscientiousness (about 1 point) there was a 9% decreased hazard of dying.

A standard deviation increase in conscientiousness was significantly associated with an 18% decreased odds of being a current smoker (path a1) and in turn, being a current smoker was associated with a 43% increased hazard of dying (path b1) over the 18-year follow-up period

8. How precise are the results?

HINT:
- look for the range of the confidence intervals, if given

Comments: Total effects are small within the study.

Total C effect on mortality = 0.84 [0.74–0.96]
Simialrly impact of C on being a current smoker is small but sig = 0.82 [0.67-0.94]

The confidence intervals within this research are large with potentially very small effects however due to the outcome metric being mortality this is still valuable information.

9. Do you believe the results?

Yes ✔
Can't Tell ☐
No ☐

HINT: Consider
- big effect is hard to ignore
- can it be due to bias, chance or confounding
- are the design and methods of this study sufficiently flawed to make the results unreliable
- Bradford Hills criteria (e.g. time sequence, dose-response gradient, biological plausibility, consistency)

Comments: Results of this study show significant results and indicate that personality to mortality relationship is mediated by how conscientiousness impacts on smoking habits.

Section C: Will the results help locally?

10. Can the results be applied to the local population?

Yes ☐
Can't Tell ☐
No ✔

HINT: Consider whether
- a cohort study was the appropriate method to answer this question
- the subjects covered in this study could be sufficiently different from your population to cause concern
- your local setting is likely to differ much from that of the study
- you can quantify the local benefits and harms

Comments: No

11. Do the results of this study fit with other available evidence?

Yes ✔
Can't Tell ☐
No ☐

Comments: This study brings together our knowledge of the impact of personality traits on both longevity and personalities impact on certain health behaviour (smoking in this case).

12. What are the implications of this study for practice?

Yes	☐
Can't Tell	☑
No	☐

HINT: Consider
- one observational study rarely provides sufficiently robust evidence to recommend changes to clinical practice or within health policy decision making
- for certain questions, observational studies provide the only evidence
- recommendations from observational studies are always stronger when supported by other evidence

Comments: This area of research is too early for it to have impacts on clinical health practise.

CASP
Critical Appraisal Skills Programme

www.casp-uk.net
info@casp-uk.net
Summertown Pavilion, Middle Way Oxford OX2 7LG

CASP Checklist: 12 questions to help you make sense of a Cohort Study

How to use this appraisal tool: Three broad issues need to be considered when appraising a cohort study:

- Are the results of the study valid? (Section A)
- What are the results? (Section B)
- Will the results help locally? (Section C)

The 12 questions on the following pages are designed to help you think about these issues systematically. The first two questions are screening questions and can be answered quickly. If the answer to both is "yes", it is worth proceeding with the remaining questions. There is some degree of overlap between the questions, you are asked to record a "yes", "no" or "can't tell" to most of the questions. A number of italicised prompts are given after each question. These are designed to remind you why the question is important. Record your reasons for your answers in the spaces provided.

About: These checklists were designed to be used as educational pedagogic tools, as part of a workshop setting, therefore we do not suggest a scoring system. The core CASP checklists (randomised controlled trial & systematic review) were based on JAMA 'Users' guides to the medical literature 1994 (adapted from Guyatt GH, Sackett DL, and Cook DJ), and piloted with health care practitioners.

For each new checklist, a group of experts were assembled to develop and pilot the checklist and the workshop format with which it would be used. Over the years overall adjustments have been made to the format, but a recent survey of checklist users reiterated that the basic format continues to be useful and appropriate.

Referencing: we recommend using the Harvard style citation, i.e.: *Critical Appraisal Skills Programme (2018). CASP (insert name of checklist i.e. Cohort Study) Checklist. [online] Available at: URL. Accessed: Date Accessed.*

©CASP this work is licensed under the Creative Commons Attribution – Non-Commercial-Share A like. To view a copy of this license, visit http://creativecommons.org/licenses/by-nc-sa/3.0/ www.casp-uk.net

Critical Appraisal Skills Programme

Paper for appraisal and reference: Smoking mediates the effect of conscientiousness on mor

Section A: Are the results of the study valid?

1. Did the study address a clearly focused issue?

Yes ☑ Can't Tell ☐ No ☐

HINT: A question can be 'focused' in terms of
- the population studied
- the risk factors studied
- is it clear whether the study tried to detect a beneficial or harmful effect
- the outcomes considered

Comments: This paper focuses directly on the question at hand and provides evidence towards smoking being a mediator of the C - Mortality relationship.

2. Was the cohort recruited in an acceptable way?

Yes ☑ Can't Tell ☐ No ☐

HINT: Look for selection bias which might compromise the generalisability of the findings:
- was the cohort representative of a defined population
- was there something special about the cohort
- was everybody included who should have been

Comments: Cohort comes from the The Veterans Affairs Normative Aging Study, a large cohort study primarily gathered to do research on aging. This paper uses a subsection of 1349 participants who completed personality measures. This cohort is acceptable in terms of size and recruitment.

Note that the cohort is only male and from a military veteran population.

Is it worth continuing?

3. Was the exposure accurately measured to minimise bias?

- [x] Yes
- [ ] Can't Tell
- [ ] No

HINT: Look for measurement or classification bias:
- did they use subjective or objective measurements
- do the measurements truly reflect what you want them to (have they been validated)
- were all the subjects classified into exposure groups using the same procedure

Comments: due to the nature of the studies there was not an exposure. Answering this question I will refer to measurement of personality and other variables of note:

Personality: Measured using Goldberg adjetive marker big 5 test

education: measured as a continous variable with detailed input ranging from primary school to college degree
Smoking was recorded and split into 3 catagories: Former, current and non smoker

A

4. Was the outcome accurately measured to minimise bias?

- [x] Yes
- [ ] Can't Tell
- [ ] No

HINT: Look for measurement or classification bias:
- did they use subjective or objective measurements
- do the measurements truly reflect what you want them to (have they been validated)
- has a reliable system been established for detecting all the cases (for measuring disease occurrence)
- were the measurement methods similar in the different groups
- were the subjects and/or the outcome assessor blinded to exposure (does this matter)

Comments: Periodic contacting is utilized to get information about mortality and then death certificates are collected for conformation of date.

CASP
Critical Appraisal
Skills Programme

5. (a) Have the authors identified all important confounding factors?

Yes ✔
Can't Tell
No

HINT:
- list the ones you think might be important, and ones the author missed

Comments: Age and education are accounted for. Gender is not applicable.

Race is not included in the study as a variable.

5. (b) Have they taken account of the confounding factors in the design and/or analysis?

Yes ✔
Can't Tell
No

HINT:
- look for restriction in design, and techniques e.g. modelling, stratified-, regression-, or sensitivity analysis to correct, control or adjust for confounding factors

Comments:

6. (a) Was the follow up of subjects complete enough?

Yes ✔
Can't Tell
No

HINT: Consider
- the good or bad effects should have had long enough to reveal themselves
- the persons that are lost to follow-up may have different outcomes than those available for assessment
- in an open or dynamic cohort, was there anything special about the outcome of the people leaving, or the exposure of the people entering the cohort

6. (b) Was the follow up of subjects long enough?

Yes ✔
Can't Tell
No

Comments: 17 year follow up with 547 (46%) of cohort having died.

Section B: What are the results?

7. What are the results of this study?

HINT: Consider
- what are the bottom line results
- have they reported the rate or the proportion between the exposed/unexposed, the ratio/rate difference
- how strong is the association between exposure and outcome (RR)
- what is the absolute risk reduction (ARR)

Comments: proportional hazards analyses confirmed that conscientiousness predicted mortality risk in an unadjusted model (HR = 0.84; CI = 0.77–0.91; p < .001). Adjusting for age, education, and self-rated health reduced the direct effect of conscientiousness but it was still significant (HR = 0.91; CI = 0.84–0.99; p < 0.05). For a standard deviation increase in conscientiousness (about 1 point) there was a 9% decreased hazard of dying.

A standard deviation increase in conscientiousness was significantly associated with an 18% decreased odds of being a current smoker (path a1) and in turn, being a current smoker was associated with a 43% increased hazard of dying (path b1) over the 18-year follow-up period

8. How precise are the results?

HINT:
- look for the range of the confidence intervals, if given

Comments: Total effects are small within the study.

Total C effect on mortality = 0.84 [0.74–0.96]
Simialrly impact of C on being a current smoker is small but sig = 0.82 [0.67-0.94]

The confidence intervals within this research are large with potentially very small effects however due to the outcome metric being mortality this is still valuable information.

CASP
Critical Appraisal
Skills Programme

9. Do you believe the results?

Yes ✔
Can't Tell ☐
No ☐

HINT: Consider
- big effect is hard to ignore
- can it be due to bias, chance or confounding
- are the design and methods of this study sufficiently flawed to make the results unreliable
- Bradford Hills criteria (e.g. time sequence, dose-response gradient, biological plausibility, consistency)

Comments: Results of this study show significant results and indicate that personality to mortality relationship is mediated by how conscientiousness impacts on smoking habits.

Section C: Will the results help locally?

10. Can the results be applied to the local population?

Yes ☐
Can't Tell ☐
No ✔

HINT: Consider whether
- a cohort study was the appropriate method to answer this question
- the subjects covered in this study could be sufficiently different from your population to cause concern
- your local setting is likely to differ much from that of the study
- you can quantify the local benefits and harms

Comments: No

11. Do the results of this study fit with other available evidence?

Yes ✔
Can't Tell ☐
No ☐

Comments: This study brings together our knowledge of the impact of personality traits on both longevity and personalities impact on certain health behaviour (smoking in this case).

12. What are the implications of this study for practice?

Yes	☐
Can't Tell	☑
No	☐

HINT: Consider
- one observational study rarely provides sufficiently robust evidence to recommend changes to clinical practice or within health policy decision making
- for certain questions, observational studies provide the only evidence
- recommendations from observational studies are always stronger when supported by other evidence

Comments: This area of research is too early for it to have impacts on clinical health practise.

CASP
Critical Appraisal Skills Programme

www.casp-uk.net
info@casp-uk.net
Summertown Pavilion, Middle Way Oxford OX2 7LG

CASP Checklist: 12 questions to help you make sense of a Cohort Study

How to use this appraisal tool: Three broad issues need to be considered when appraising a cohort study:

- Are the results of the study valid? (Section A)
- What are the results? (Section B)
- Will the results help locally? (Section C)

The 12 questions on the following pages are designed to help you think about these issues systematically. The first two questions are screening questions and can be answered quickly. If the answer to both is "yes", it is worth proceeding with the remaining questions. There is some degree of overlap between the questions, you are asked to record a "yes", "no" or "can't tell" to most of the questions. A number of italicised prompts are given after each question. These are designed to remind you why the question is important. Record your reasons for your answers in the spaces provided.

About: These checklists were designed to be used as educational pedagogic tools, as part of a workshop setting, therefore we do not suggest a scoring system. The core CASP checklists (randomised controlled trial & systematic review) were based on JAMA 'Users' guides to the medical literature 1994 (adapted from Guyatt GH, Sackett DL, and Cook DJ), and piloted with health care practitioners.

For each new checklist, a group of experts were assembled to develop and pilot the checklist and the workshop format with which it would be used. Over the years overall adjustments have been made to the format, but a recent survey of checklist users reiterated that the basic format continues to be useful and appropriate.

Referencing: we recommend using the Harvard style citation, i.e.: *Critical Appraisal Skills Programme (2018). CASP (insert name of checklist i.e. Cohort Study) Checklist. [online] Available at: URL. Accessed: Date Accessed.*

©CASP this work is licensed under the Creative Commons Attribution – Non-Commercial-Share A like. To view a copy of this license, visit http://creativecommons.org/licenses/by-nc-sa/3.0/ www.casp-uk.net

CASP
Critical Appraisal
Skills Programme

Paper for appraisal and reference: Smoking mediates the effect of conscientiousness on mor

Section A: Are the results of the study valid?

1. Did the study address a clearly focused issue?

Yes ✔
Can't Tell ☐
No ☐

HINT: A question can be 'focused' in terms of
- the population studied
- the risk factors studied
- is it clear whether the study tried to detect a beneficial or harmful effect
- the outcomes considered

Comments: This paper focuses directly on the question at hand and provides evidence towards smoking being a mediator of the C - Mortality relationship.

2. Was the cohort recruited in an acceptable way?

Yes ✔
Can't Tell ☐
No ☐

HINT: Look for selection bias which might compromise the generalisability of the findings:
- was the cohort representative of a defined population
- was there something special about the cohort
- was everybody included who should have been

Comments: Cohort comes from the The Veterans Affairs Normative Aging Study, a large cohort study primarily gathered to do research on aging. This paper uses a subsection of 1349 participants who completed personality measures. This cohort is acceptable in terms of size and recruitment.

Note that the cohort is only male and from a military veteran population.

Is it worth continuing?

3. Was the exposure accurately measured to minimise bias?

Yes ☑
Can't Tell ☐
No ☐

HINT: Look for measurement or classification bias:
- did they use subjective or objective measurements
- do the measurements truly reflect what you want them to (have they been validated)
- were all the subjects classified into exposure groups using the same procedure

Comments: due to the nature of the studies there was not an exposure. Answering this question I will refer to measurement of personality and other variables of note:

Personality: Measured using Goldberg adjetive marker big 5 test

education: measured as a continous variable with detailed input ranging from primary school to college degree
Smoking was recorded and split into 3 catagories: Former, current and non smoker

A

4. Was the outcome accurately measured to minimise bias?

Yes ☑
Can't Tell ☐
No ☐

HINT: Look for measurement or classification bias:
- did they use subjective or objective measurements
- do the measurements truly reflect what you want them to (have they been validated)
- has a reliable system been established for detecting all the cases (for measuring disease occurrence)
- were the measurement methods similar in the different groups
- were the subjects and/or the outcome assessor blinded to exposure (does this matter)

Comments: Periodic contacting is utilized to get information about mortality and then death certificates are collected for conformation of date.

5. (a) Have the authors identified all important confounding factors?

Yes ✔
Can't Tell
No

HINT:
- list the ones you think might be important, and ones the author missed

Comments: Age and education are accounted for. Gender is not applicable.

Race is not included in the study as a variable.

5. (b) Have they taken account of the confounding factors in the design and/or analysis?

Yes ✔
Can't Tell
No

HINT:
- look for restriction in design, and techniques e.g. modelling, stratified-, regression-, or sensitivity analysis to correct, control or adjust for confounding factors

Comments:

6. (a) Was the follow up of subjects complete enough?

Yes ✔
Can't Tell
No

HINT: Consider
- the good or bad effects should have had long enough to reveal themselves
- the persons that are lost to follow-up may have different outcomes than those available for assessment
- in an open or dynamic cohort, was there anything special about the outcome of the people leaving, or the exposure of the people entering the cohort

6. (b) Was the follow up of subjects long enough?

Yes ✔
Can't Tell
No

Comments: 17 year follow up with 547 (46%) of cohort having died.

Section B: What are the results?

7. What are the results of this study?

HINT: Consider
- what are the bottom line results
- have they reported the rate or the proportion between the exposed/unexposed, the ratio/rate difference
- how strong is the association between exposure and outcome (RR)
- what is the absolute risk reduction (ARR)

Comments: proportional hazards analyses confirmed that conscientiousness predicted mortality risk in an unadjusted model (HR = 0.84; CI = 0.77–0.91; p < .001). Adjusting for age, education, and self-rated health reduced the direct effect of conscientiousness but it was still significant (HR = 0.91; CI = 0.84–0.99; p < 0.05). For a standard deviation increase in conscientiousness (about 1 point) there was a 9% decreased hazard of dying.

A standard deviation increase in conscientiousness was significantly associated with an 18% decreased odds of being a current smoker (path a1) and in turn, being a current smoker was associated with a 43% increased hazard of dying (path b1) over the 18-year follow-up period

8. How precise are the results?

HINT:
- look for the range of the confidence intervals, if given

Comments: Total effects are small within the study.

Total C effect on mortality = 0.84 [0.74–0.96]
Simialrly impact of C on being a current smoker is small but sig = 0.82 [0.67-0.94]

The confidence intervals within this research are large with potentially very small effects however due to the outcome metric being mortality this is still valuable information.

9. Do you believe the results?

Yes	✔
Can't Tell	
No	

HINT: Consider
- big effect is hard to ignore
- can it be due to bias, chance or confounding
- are the design and methods of this study sufficiently flawed to make the results unreliable
- Bradford Hills criteria (e.g. time sequence, dose-response gradient, biological plausibility, consistency)

Comments: Results of this study show significant results and indicate that personality to mortality relationship is mediated by how conscientiousness impacts on smoking habits.

Section C: Will the results help locally?

10. Can the results be applied to the local population?

Yes	
Can't Tell	
No	✔

HINT: Consider whether
- a cohort study was the appropriate method to answer this question
- the subjects covered in this study could be sufficiently different from your population to cause concern
- your local setting is likely to differ much from that of the study
- you can quantify the local benefits and harms

Comments: No

11. Do the results of this study fit with other available evidence?

Yes	✔
Can't Tell	
No	

Comments: This study brings together our knowledge of the impact of personality traits on both longevity and personalities impact on certain health behaviour (smoking in this case).

CASP
Critical Appraisal
Skills Programme

12. What are the implications of this study for practice?

Yes ☐
Can't Tell ☑
No ☐

HINT: Consider
- one observational study rarely provides sufficiently robust evidence to recommend changes to clinical practice or within health policy decision making
- for certain questions, observational studies provide the only evidence
- recommendations from observational studies are always stronger when supported by other evidence

Comments: This area of research is too early for it to have impacts on clinical health practise.
